# Volumes 30 and 31 Index

**Published:** 2008

## Author Index

### A

Adams, C.A.

On the Use of Short-Interfering RNA to Study

  Alcohol-Related Genes

  Vol. 31, No. 3, Pages 256–258

Agrawal, A.

  The Genetics of Alcohol and Other Drug Dependence

  Vol. 31, No. 2, Pages 111–118

Apte, M.V.

  Role of Alcohol Metabolism in Chronic Pancreatitis

  Vol. 30, No. 1, Pages 48–54

Arias, A.J.

  Treatment of Co-Occurring

  Alcohol and Other Drug Use Disorders

  Vol. 31, No. 2, Pages 155–167

Arnaout, B.

  Diagnosing Co-Morbid Drug Use in Patients

  With Alcohol Use Disorders

  Vol. 31, No. 2, Pages 148–154

### B

Badger, T.M.

  Effects of Pregnancy and Nutritional Status on Alcohol Metabolism

  Vol. 30, No. 1, Pages 55–59

Bajo, M.

  Shared Mechanisms of Alcohol and Other Drugs

  Vol. 31, No. 2, Pages 137–147

Becker, H.

  Alcohol Dependence, Withdrawal, and Relapse

  Vol. 31, No. 4, Pages 343–361

Becker, P.

  Alcohol Metabolism and Cancer Risk

  Vol. 30, No. 1, Pages 38–47

Belknap, J.K.

  Quantitative Trait Locus Analysis: Multiple

  Cross and Heterogeneous Stock Mapping

  Vol. 31, No. 3, Pages 261–265

  Single-Nucleotide Polymorphism Masking

  Vol. 31, No. 3, Pages 270–271

Bhave, S.V.

  Expression Quantitative Trait Loci and the PhenoGen Database

  Vol. 31, No. 3, Pages 272–274

  How Adaptations of the Brain to Alcohol Leads to Dependence: A Pharmacological Perspective

  Vol. 31, No. 4, Pages 310–339

Boros, L.

  Metabolomics in Alcohol Research and Drug Development

  Vol. 31, No. 1, Pages 26–35

Boudreau, E.A.

  Magnetic Resonance Imaging Approaches for Studying Alcoholism Using Mouse Models

  Vol. 31, No. 3, Pages 247–248

Buck, K.J.

  Interval-Specific Congenic Animals for High-Resolution Quantitative Trait Loci Mapping

  Vol. 31, No. 3, Pages 266–269

  Single-Nucleotide Polymorphism Masking

  Vol. 31, No. 3, Pages 270–271

### C

Carr, L.G.

  Variations in Alcohol-Metabolizing Enzymes in People of East Indian and African Descent

  From Trinidad and Tobago

  Vol. 30, No. 1, Pages 28–30

Chen, G.

  Magnetic Resonance Imaging Approaches for Studying Alcoholism Using Mouse Models

  Vol. 31, No. 3, Pages 247–248

Clapp, P.

  How Adaptation of the Brain to Alcohol Leads to Dependence: A Pharmacological Perspective

  Vol. 31, No. 4, Pages 310–339

Clark, D.B.

  Adolescents at Risk for Substance Use

  Disorders

  Vol. 31, No. 2, Pages 168–176

Crews, F.T.

  Alcohol-Related Neurodegeneration and Recovery: Mechanisms From Animal Models

  Vol. 31, No. 4, Pages 377–388

Cruz, M.T.

  Shared Mechanisms of Alcohol and Other Drugs

  Vol. 31, No. 2, Pages 137–147

## D

Denmark, D.L.

  Interval-Specific Congenic Animals for High-Resolution Quantitative Trait Loci Mapping

  Vol. 31, No. 3, Pages 266–269

Dick, D.M.

  The Genetics of Alcohol and Other Drug Dependence

  Vol. 31, No. 2, Pages 111–118

### E

Edenberg, H.J.

  The Genetics of Alcohol Metabolism:

  Role of Alcohol Dehydrogenase and Aldehyde

  Dehydrogenase Variants

  Vol. 30, No. 1, Pages 5–13

Ehlers, C.L.

  Variations in ADH and ALDH in Southwest

  California Indians

  Vol. 30, No. 1, Pages 14–17

  Variations in Alcohol-Metabolizing Enzymes in People of East Indian and African Descent

  From Trinidad and Tobago

  Vol. 30, No. 1, Pages 28–30

Eng, M.Y.

  *ALDH2, ADH1B,* and *ADH1C* Genotypes in Asians: A Literature Review

  Vol. 30, No. 1, Pages 22–27

### F

Falk, D.

  An Epidemiologic Analysis of Co-Occurring

  Alcohol and Other Drug Use and Disorders

  Vol. 31, No. 2, Pages 100–110

Faustman E.M.

  A Systems-Based Computational Model of Alcohol’s Toxic Effects on Brain Development

  Vol. 31, No. 1, Pages 76–83

Ferguson, B.

  Who Is at Risk? Self-Administration in Nonhuman Primates Helps Identify Pathways to Dependence

  Vol. 31, No. 4, Pages 289–297

Fischer, H.P.

  Mathematical Modeling of Complex Biological

  Systems: From Parts Lists to Understanding

  Systems Behavior

  Vol. 31, No. 1, Pages 49–59

### G

Gilbertson, R.

  The Role of Selected Factors in the Development and Consequences of Alcohol

  Dependence

  Vol. 31, No. 4, Pages 389–399

Gohlke, J.M.

  A Systems-Based Computational Model of Alcohol’s Toxic Effects on Brain Development

  Vol. 31, No. 1, Pages 76–83

Grant, K.

  Who Is at Risk? Self-Administration in Nonhuman Primates Helps Identify

  Pathways to Dependence

  Vol. 31, No. 4, Pages 289–297

Gilpin, N.W.

  Neurobiology of Alcohol Dependence: Focus on Motivational Mechanisms

  Vol. 31, No. 3, Pages 185–195

Guidot, D.M.

  Alcoholic Lung Disease

  Vol. 31, No. 1, Pages 66–75

Guo, Q.M.

  Commentary: Systems Biology and Its Relevance to Alcohol Research

  Vol. 31, No. 1, Pages 5–10

## H

Harrigan, G.G.

  Metabolomics in Alcohol Research and Drug

  Development

  Vol. 31, No. 1, Pages 26–35

Heberlein, U.

  Viral Delivery of Small-Hairpin RNAs for Reducing Gene Expression in the Rodent Brain

  Vol. 31, No. 3, Pages 259–260

Hiller-Sturmhöfel, S.

  Proteomic Approaches for Studying Alcoholism and Alcohol-Induced Organ Damage

  Vol. 31, No. 1, Pages 36–48

  Systems Biology in the Study of Neurological

  Disorders: Focus on Alzheimer’s Disease

  Vol. 31, No. 1, Pages 60–65

  A Systems-Based Computational Model of Alcohol’s Toxic Effects on Brain Development

  Vol. 31, No. 1, Pages 76–83

  An Epidemiologic Analysis of Co-Occurring

  Alcohol and Other Drug Use and Disorders

  Vol. 31, No. 2, Pages 100–110

Hitzemann, R.

  Strategies to Study the Neuroscience of Alcoholism: Introduction

  Vol. 31, No. 3, Pages 231–232

  Quantitative Trait Locus Analysis: Multiple

  Cross and Heterogeneous Stock Mapping

  Vol. 31, No. 3, Pages 261–265

  Single-Nucleotide Polymorphism Masking

  Vol. 31, No. 3, Pages 270–271

  Conclusions

  Vol. 31, No. 3, Page 278

Hoffman, P.L.

  Expression Quantitative Trait Loci and the PhenoGen Database

  Vol. 31, No. 3, Pages 272–274

  How Adaptation of the Brain to Alcohol Leads to Dependence: A Pharmacological Perspective

  Vol. 31, No. 4, Pages 310–339

Hornbaker, C.

  Expression Quantitative Trait Loci and the PhenoGen Database

  Vol. 31, No. 3, Pages 272–274

### K

Kantor, L.W.

  NIH Roadmap for Medical Research

  Vol. 31, No. 1, Pages 12–13

Kershaw, C.D.

  Alcoholic Lung Disease

  Vol. 31, No. 1, Pages 66–75

Kiley, C.

  Who Is at Risk? Self-Administration in Nonhuman Primates Helps Identify Pathways to Dependence

  Vol. 31, No. 4, Pages 289–297

Koob, G.F.

  Neurobiology of Alcohol Dependence: Focus on Motivational Mechanisms

  Vol. 31, No. 3, Pages 185–195

Kranzler, H.R.

  What Is Addiction?

  Vol. 31, No. 2, Pages 93–95

  Treatment of Co-Occurring Alcohol and Other Drug Use Disorders

  Vol. 31, No. 2, Pages 155–167

Krishnan-Sarin, S.

  Treatment Implications: Using Neuroscience to Guide the Development of New

  Pharmacotherapies for Alcoholism

  Vol. 31, No. 4, Pages 400–407

Kroenke, C.D.

  The Use of Magnetic Resonance Spectroscopy and Magnetic Resonance Imaging in Alcohol

  Research

  Vol. 31, No. 3, Pages 243–246

  Magnetic Resonance Imaging Approaches for Studying Alcoholism Using Mouse Models

  Vol. 31, No. 3, Pages 247–248

Krystal, J.H.

  Treatment Implications: Using Neuroscience to Guide the Development of New

  Pharmacotherapies for Alcoholism

  Vol. 31, No. 4, Pages 400–407

### L

Lasek, A.W.

  Viral Delivery of Small-Harpin RNAs for Reducing Gene Expression in the Rodent Brain

  Vol. 31, No. 3, Pages 259–260

Li, T.-K.

  What Is Addiction?

  Vol. 31, No. 2, Pages 93–95

Li, X.

  Magnetic Resonance Imaging Approaches for Studying Alcoholism Using Mouse Models

  Vol. 31, No. 3, Pages 247–248

Lovinger, D.M.

  Communication Networks in the Brain:

  Neurons, Receptors, Neurotransmitters, and Alcohol

  Vol. 31, No. 3, Pages 196–214

Lu, L.

  Integrative Genetic Analysis of Alcohol

  Dependence Using the GeneNetwork

  Web Resources

  Vol. 31, No. 3, Pages 275–277

Luczak, S.E.

  *ALDH2, ADH1B,* and *ADH1C* Genotypes in Asians: A Literature Review

  Vol. 30, No. 1, Pages 22–27

### M

MacCoss, M.J.

  Proteomic Solutions for Analytical Challenges

  Associated With Alcohol Research

  Vol. 31, No. 3, Pages 251–255

Maguire, G.

  Metabolomics in Alcohol Research and Drug Development

  Vol. 31, No. 1, Pages 26–35

Martin, C.S.

  Timing of Alcohol and Other Drug Use

  Vol. 31, No. 2, Pages 96–99

Mayfield, R.D.

  Proteomic Approaches for Studying Alcoholism and Alcohol-Induced Organ Damage

  Vol. 31, No. 1, Pages 36–48

McWeeney, S.K.

  Quantitative Trait Locus Analysis: Multiple

  Cross and Heterogeneous Stock Mapping

  Vol. 31, No. 3, Pages 261–265

  Single-Nucleotide Polymorphism Masking

  Vol. 31, No. 3, Pages 270–271

Milner, L.C.

  Interval-Specific Congenic Animals for High-Resolution Quantitative Trait Loci Mapping

  Vol. 31, No. 3, Pages 266–269

Montane-Jaime, L.K.

  Variations in Alcohol-Metabolizing Enzymes in People of East Indian and African Descent

  From Trinidad and Tobago

  Vol. 30, No. 1, Pages 28–30

Moore, J.H.

  Systems Genetics of Alcoholism

  Vol. 31, No. 1, Pages 14–25

Moore, S.

  Variations in Alcohol-Metabolizing Enzymes in People of East Indian and African Descent

  From Trinidad and Tobago

  Vol. 30, No. 1, Pages 28–30

### N

Nagel, B.J.

  The Use of Magnetic Resonance

  Spectroscopy and Magnetic Resonance

  Imaging in Alcohol Research

  Vol. 31, No. 3, Pages 243–246

Nixon, S.J.

  The Role of Selected Factors in the Development and Consequences of Alcohol Dependence

  Vol. 31, No. 4, Pages 389–399

### O

Oberbeck, D.

  Strategies to Study the Neuroscience of Alcoholism: Introduction

  Vol. 31, No. 3, Pages 231–232

  Laser-Assisted Microdissection

  Vol. 31, No. 3, Pages 249–250

  Conclusions

  Vol. 31, No. 3, Page 278

Odagiri, M.

  Who Is at Risk? Self-Administration in Nonhuman Primates Helps Identify Pathways to Dependence

  Vol. 31, No. 4, Pages 289–297

O’Malley, S.

  Treatment Implications: Using Neuroscience to Guide the Development of New

  Pharmacotherapies for Alcoholism

  Vol. 31, No. 4, Pages 400–407

### P

Pasinetti, G.M.

  Systems Biology in the Study of Neurological

  Disorders: Focus on Alzheimer’s Disease

  Vol. 31, No. 1, Pages 60–65

Peters, S.T.

  Single-Nucleotide Polymorphism Masking

  Vol. 31, No. 3, Pages 270–271

Petrakis, I.L.

  Diagnosing Co-Morbid Drug Use in Patients

  With Alcohol Use Disorders

  Vol. 31, No. 2, Pages 148–154

Pietrzykowski, A.Z.

  The Molecular Basis of Tolerance

  Vol. 31, No. 4, Pages 298–309

Pfefferbaum, A.

  Magnetic Resonance Imaging of the Living

  Brain: Evidence for Brain Degeneration

  Among Alcoholics and Recovery With Abstinence

  Vol. 31, No. 4, Pages 369–376

Pirola, R.C.

  Role of Alcohol Metabolism in Chronic Pancreatitis

  Vol. 30, No. 1, Pages 48–54

Porjesz, B.

  From Event-Related Potential to Oscillations:

  Genetic Diathesis in Brain (Dys)Function and Alcohol Dependence

  Vol. 31, No. 3, Pages 238–242

Prather, R.

  The Role of Selected Factors in the Development and Consequences of Alcohol Dependence

  Vol. 31, No. 4, Pages 389–399

### R

Rangaswamy, M.

  From Event-Related Potential to Oscillations:

  Genetic Diathesis in Brain (Dys)Function and Alcohol Dependence

  Vol. 31, No. 3, Pages 238–242

Roberto, M.

  Shared Mechanisms of Alcohol and Other Drugs

  Vol. 31, No. 2, Pages 137–147

Ronis, M.J.J.

  Effects of Pregnancy and Nutritional

  Status on Alcohol Metabolism

  Vol. 30, No. 1, Pages 55–59

Rosenbloom, M.J.

  Magnetic Resonance Imaging of the Living

  Brain: Evidence for Brain Degeneration

  Among Alcoholics and Recovery With Abstinence

  Vol. 31, No. 4, Pages 369–376

### S

Saba, L.

  Expression Quantitative Trait Loci and the PhenoGen Database

  Vol. 31, No. 3, Pages 272–274

Sayarath, V.

  Systems Genetics of Alcoholism

  Vol. 31, No. 1, Pages 14–25

Schweitzer, P.

  Shared Mechanisms of Alcohol and Other Drugs

  Vol. 31, No. 2, Pages 137–147

Scott, D.M.

  Health-Related Effects of Genetic Variations of Alcohol-Metabolizing Enzymes in African

  Americans

  Vol. 30, No. 1, Pages 18–21

Seitz, H.K.

  Alcohol Metabolism and Cancer Risk

  Vol. 30, No. 1, Pages 38–47

Shankar, K.

  Effects of Pregnancy and Nutritional Status on Alcohol Metabolism

  Vol. 30, No. 1, Pages 55–59

Sloan, C.D.

  Systems Genetics of Alcoholism

  Vol. 31, No. 1, Pages 14–25

Stafford, J.

  Who Is at Risk? Self-Administration in Nonhuman Primates Helps Identify Pathways to Dependence

  Vol. 31, No. 4, Pages 289–297

Sobin, J.

  Proteomic Approaches for Studying Alcoholism and Alcohol-Induced Organ Damage

  Vol. 31, No. 1, Pages 36–48

Sullivan, E.V.

  Translational Studies of Alcoholism: Bridging the Gap

  Vol. 31, No. 3, Pages 215–230

### T

Tabakoff, B.

  Expression Quantitative Trait Loci and the PhenoGen Database

  Vol. 31, No. 3, Pages 272–274

Taylor, R.E.

  Health-Related Effects of Genetic Variations of Alcohol-Metabolizing Enzymes in African

  Americans

  Vol. 30, No. 1, Pages 18–21

Thanos, P.K.

  Positron Emission Tomography As a Tool for Studying Alcohol Abuse

  Vol. 31, No. 3, Pages 233–237

Thatcher, D.L.

  Adolescents at Risk for Substance

  Use Disorders

  Vol. 31, No. 2, Pages 168–176

Theide, A.

  Who Is at Risk? Self-Administration in Nonhuman Primates Helps Identify Pathways to Dependence

  Vol. 31, No. 4, Pages 289–297

Treistman, S.N.

  The Molecular Basis of Tolerance

  Vol. 31, No. 4, Pages 289–309

### V

Volkow, N.D.

  Positron Emission Tomography As a Tool for Studying Alcohol Abuse

  Vol. 31, No. 3, Pages 233–237

Vonlaufen, A.

  of Alcohol Metabolism in Chronic Pancreatitis

  Vol. 30, No. 1, Pages 48–54

### W

Wall, T.L.

  *ALDH2, ADH1B,* and *ADH1C* Genotypes in Asians: A Literature Review

  Vol. 30, No. 1, Pages 22–27

Walter, N.A.R.

  Single-Nucleotide Polymorphism Masking

  Vol. 31, No. 3, Pages 270–271

Wand, G.

  The Influence of Stress on the Transition From

  Drug Use to Addiction

  Vol. 31, No. 2, Pages 119–136

Wang, G.-J.

  Positron Emission Tomography As a Tool for Studying Alcohol Abuse

  Vol. 31, No. 3, Pages 233–237

Williams, R.W.

  Integrative Genetic Analysis of Alcohol

  Dependence Using the GeneNetwork

  Web Resources

  Vol. 31, No. 3, Pages 275–277

Wilson, J.S.

  Role of Alcohol Metabolism in Chronic Pancreatitis

  Vol. 30, No. 1, Pages 48–54

Wu, C.C.

  Proteomic Solutions for Analytical Challenges

  Associated With Alcohol Research

  Vol. 31, No. 3, Pages 251–255

### Y

Yi, H.-Y.

  An Epidemiologic Analysis of Co-Occurring

  Alcohol and Other Drug Use and Disorders

  Vol. 31, No. 2, Pages 100–110

Yin, H.H.

  From Actions to Habits: Neuroadaptations

  Leading to Dependence

  Vol. 31, No. 4, Pages 340–347

### Z

Zahr, N.M.

  Translational Studies of Alcoholism:

  Bridging the Gap

  Vol. 31, No. 3, Pages 215–230

Zakhari, S.

  Commentary: Systems Biology and Its

  Relevance to Alcohol Research

  Vol. 31, No. 1, Pages 5–10

Zawada, W.M.

  On the Use of Short-Interfering RNA to Study Alcohol-Related Genes

  Vol. 31, No. 3, Pages 256–258

## Index of Articles

### A

Adolescents at Risk for Substance Use Disorders

  D.L. Thatcher and D.B. Clark

  Vol. 31, No. 2, Pages 168–176

Alcohol Dependence, Withdrawal, and Relapse

  H.C. Becker

  Vol. 31, No. 4, Pages 348–361

Alcoholic Lung Disease

  C.D. Kershaw and D.M. Guidot

  Vol. 31, No. 1, Pages 66–75

Alcohol Metabolism and Cancer Risk

  H.K. Seitz and P. Becker

  Vol. 30, No. 1, Pages 38–47

Alcohol-Related Neurodegeneration and Recovery:

  Mechanisms From Animal Models

  F.T. Crews

  Vol. 31, No. 4, Pages 377–388

*ALDH2, ADH1B*, and *ADH1C* Genotypes in Asians: A Literature Review

  M.Y. Eng, S.E. Luczak, and T.L. Wall

  Vol. 30, No. 1, Pages 22–27

### C

Commentary: Systems Biology and Its Relevance to Alcohol Research

  Q.M. Guo and S. Zakhari

  Vol. 31, No. 1, Pages 5–10

Communication Networks in the Brain: Neurons, Receptors, Neurotransmitters, and Alcohol

  D.M. Lovinger

  Vol. 31, No. 3, Pages 196–214

### D

Diagnosing Co-Morbid Drug Use in Patients With

  Alcohol Use Disorders

  B. Arnaout and I.L. Petrakis

  Vol. 31, No. 2, Pages 148–154

### E

Effects of Pregnancy and Nutritional Status on Alcohol Metabolism

  K. Shankar, M.J.J. Ronis, and T.M. Badger

  Vol. 30, No. 1, Pages 55–59

An Epidemiologic Analysis of Co-Occurring

  Alcohol and Other Drug Use and Disorders

  D. Falk, H.-Y. Yi, and S. Hiller-Sturmhöfel

  Vol. 31, No. 2, Pages 100–110

Expression Quantitative Trait Loci and the PhenoGen Database

  L. Saba, P.L. Hoffman, C. Hornbaker, S.V. Bhave, and B. Tabakoff

  Vol. 31, No. 3, Pages 272–274

### F

From Actions to Habits: Neuroadaptations

  Leading to Dependence

  H.H. Yin

  Vol. 31, No. 4, Pages 340–347

From Event-Related Potential to Oscillations:

  Genetic Diathesis in Brain (Dys)Function and Alcohol Dependence

  M. Rangaswamy and B. Porjesz

  Vol. 31, No. 3, Pages 238–242

### G

The Genetics of Alcohol and Other Drug

  Dependence

  D.M. Dick and A. Agrawal

  Vol. 31, No. 2, Pages 111–118

The Genetics of Alcohol Metabolism:

  Role of Alcohol Dehydrogenase and Aldehyde

  Dehydrogenase Variants

  H.J. Edenberg

  Vol. 30, No. 1, Pages 5–13

### H

Health-Related Effects of Genetic Variations of Alcohol-Metabolizing Enzymes in African Americans

  D.M. Scott and R.E. Taylor

  Vol. 30, No. 1, Pages 18–21

How Adaptation of the Brain to Alcohol Leads to Dependence: A Pharmacological Perspective

  P. Clapp, S.V. Bhave, and P.L. Hoffman

  Vol. 31, No. 4, Pages 310–339

### I

The Influence of Stress on the Transition From

  Drug Use to Addiction

  G. Wand

  Vol. 31, No. 2, Pages 119–136

Integrative Genetic Analysis of Alcohol

  Dependence Using the GeneNetwork

  Web Resources

  R.W. Williams and L. Lu

  Vol. 31, No. 3, Pages 275–277

Interval-Specific Congenic Animals for High-Resolution Quantitative Trait Loci Mapping

  D.L. Denmark, L.C. Milner, and K.J. Buck

  Vol. 31, No. 3, Pages 266–269

### L

Laser-Assisted Microdissection

  D. Oberbeck

  Vol. 31, No. 3, Pages 249–250

### M

Magnetic Resonance Imaging Approaches for Studying Alcoholism Using Mouse Models

  E.A. Boudreau, G. Chen, X. Li, and C.D. Kroenke

  Vol. 31, No. 3, Pages 247–248

Magnetic Resonance Imaging of the Living Brain:

  Evidence for Brain Degeneration Among

  Alcoholics and Recovery With Abstinence

  M.J. Rosenbloom and A. Pfefferbaum

  Vol. 31, No. 4, Pages 362–376

Mathematical Modeling of Complex Biological

  Systems: From Parts Lists to Understanding

  Systems Behavior H.P. Fischer

  Vol. 31, No. 1, Pages 49–59

Metabolomics in Alcohol Research and Drug

  Development

  G.G. Harrigan, G. Maguire, and L. Boros

  Vol. 31, No. 1, Pages 26–35

The Molecular Basis of Tolerance

  A.Z. Pietryzkowski and S.N. Treistman

  Vol. 31, No. 4, Pages 298–309

### N

Neurobiology of Alcohol Dependence:

  Focus on Motivational Mechanisms

  N.W. Gilpin and G.F. Koob

  Vol. 31, No. 3, Pages 185–195

NIH Roadmap for Medical Research

  L.W. Kantor

  Vol. 31, No. 1, Pages 12–13

### O

On the Use of Short-Interfering RNA to Study

  Alcohol-Related Genes

  C.A. Adams and W.M. Zawada

  Vol. 31, No. 3, Pages 256–258

### P

Positron Emission Tomography As a Tool for Studying Alcohol Abuse

  P.K. Thanos, G.-J. Wang, and N.D. Volkow

  Vol. 31, No. 3, Pages 233–237

Proteomic Approaches for Studying Alcoholism and Alcohol-Induced Organ Damage

  S. Hiller-Sturmhöfel, J. Sobin, and R.D. Mayfield

  Vol. 31, No. 1, Pages 36–48

Proteomic Solutions for Analytical Challenges

  Associated With Alcohol Research

  M.J. MacCoss and C.C. Wu

  Vol. 31, No. 3, Pages 251–255

### Q

Quantitative Trait Locus Analysis: Multiple Cross and Heterogeneous Stock Mapping

  R. Hitzemann, J.K. Belknap, and S.K. McWeeney

  Vol. 31, No. 3, Pages 261–265

### R

Role of Alcohol Metabolism in Chronic

  Pancreatitis

  A. Vonlaufen, J.S. Wilson, R.C. Pirola, and M.V. Apte

  Vol. 30, No. 1, Pages 48–54

The Role of Selected Factors in the Development and Consequences of Alcohol Dependence

  R. Gilbertson, R. Prather, and S.J. Nixon

  Vol. 31, No. 4, Pages 389–399

### S

Shared Mechanisms of Alcohol and Other Drugs

  M.T. Cruz, M. Bajo, P. Schweitzer, and M. Roberto

  Vol. 31, No. 2, Pages 137–147

Single-Nucleotide Polymorphism Masking

  N.A.R. Walter, S.K. McWeeney, S.T. Peters, J.K. Belknap, R. Hitzemann, and K.J. Buck

  Vol. 31, No. 3, Pages 270–271

Strategies to Study the Neuroscience of Alcoholism: Introduction

  R. Hitzemann and D. Oberbeck

  Vol. 31, No. 3, Pages 231–232

A Systems-Based Computational Model of Alcohol’s Toxic Effects on Brain Development

  J.M. Gohlke, S. Hiller-Sturmhöfel, and E.M. Faustman

  Vol. 31, No. 1, Pages 76–83

Systems Biology in the Study of Neurological

  Disorders: Focus on Alzheimer’s Disease

  G.M. Pasinetti and S. Hiller-Sturmhöfel

  Vol. 31, No. 1, Pages 60–65

Systems Genetics of Alcoholism

  C.D. Sloan, V. Sayarath, and J. H. Moore

  Vol. 31, No. 1, Pages 14–25

### T

Timing of Alcohol and Other Drug Use

  C.S. Martin

  Vol. 31, No. 2, Pages 96–99

Translational Studies of Alcoholism:

  Bridging the Gap

  N.M. Zahr and E.V. Sullivan

  Vol. 31, No. 3, Pages 215–230

Treatment Implications: Using Neuroscience to Guide the Development of New

  Pharmacotherapies for Alcoholism

  S. Krishnan-Sarin, S. O’Malley, and J.H. Krystal

  Vol. 31, No. 4, Pages 400–407

Treatment of Co-Occurring Alcohol and Other Drug Use Disorders

  A.J. Arias and H.R. Kranzler

  Vol. 31, No. 2, Pages 155–167

### U

The Use of Magnetic Resonance Spectroscopy and Magnetic Resonance Imaging in Alcohol

  Research

  B.J. Nagel and C.D. Kroenke

  Vol. 31, No. 3, Pages 243–246

### V

Variations in ADH and ALDH in Southwest

  California Indians

  C.L. Ehlers

  Vol. 30, No. 1, Pages 14–17

Variations in Alcohol-Metabolizing Enzymes in People of East Indian and African

  Descent From Trinidad and Tobago

  S. Moore, L.K. Montane-Jaime, L.G. Carr, and C.L. Ehlers

  Vol. 30, No. 1, Pages 28–30

Viral Delivery of Small-Hairpin RNAs for Reducing Gene Expression in the Rodent Brain

  A.W. Lasek and U. Heberlein

  Vol. 31, No. 3, Pages 259–260

### W

What Is Addiction?

  H.R. Kranzler and T.-K. Li

  Vol. 31, No. 2, Pages 93–95

Who Is at Risk? Self-Administration in Nonhuman

  Primates Helps Identify Pathways to Dependence

  K. Grant, J. Stafford, A. Theide, C. Kiley, M. Odagiri, and B. Ferguson

  Vol. 31, No. 4, Pages 289–297

